# Applying for, reviewing and funding public health research in Germany and beyond

**DOI:** 10.1186/s12961-016-0112-5

**Published:** 2016-06-13

**Authors:** Ansgar Gerhardus, Heiko Becher, Peter Groenewegen, Ulrich Mansmann, Thorsten Meyer, Holger Pfaff, Milo Puhan, Oliver Razum, Eva Rehfuess, Rainer Sauerborn, Daniel Strech, Frank Wissing, Hajo Zeeb, Eva Hummers-Pradier

**Affiliations:** Department for Health Services Research, Institute for Public Health and Nursing Research, Health Sciences Bremen, University of Bremen, Bremen, Germany; Institute of Medical Biometry and Epidemiology, University Medical Center Hamburg-Eppendorf, Hamburg, Germany; Netherlands Institute for Health Services Research, Utrecht, The Netherlands; Departments of Sociology and Human Geography, Utrecht University, Utrecht, The Netherlands; Department of Medical Information Sciences, Biometrics, and Epidemiology, Ludwig-Maximilians-University, Munich, Germany; Integrative Rehabilitation Research Unit, Institute for Epidemiology, Social Medicine and Health Systems Research, Hannover Medical School, Hannover, Germany; Institute for Medical Sociology, Health Services Research and Rehabilitation Science, Center for Health Services Research Cologne, Faculty of Human Science and Faculty of Medicine, University of Cologne, Cologne, Germany; Epidemiology, Biostatistics & Prevention Institute, University of Zurich, Zurich, Switzerland; Department of Epidemiology and International Public Health, School of Public Health, Bielefeld University, Bielefeld, Germany; Institute for Public Health, Heidelberg University Hospital, Heidelberg, Germany; CELLS – Centre for Ethics and Law in the Life Sciences, Institute for History, Ethics and Philosophy in Medicine, Hannover Medical School, Hannover, Germany; German Medical Faculties’ Association (MFT), Berlin, Germany; Leibniz Institute for Prevention Research and Epidemiology, Department of Prevention and Evaluation, Health Sciences Bremen, University of Bremen, Bremen, Germany; Department of General Practice, University Medical Center Göttingen, Göttingen, Germany

**Keywords:** Public health, Research, Grants peer review, Funding, Health systems research, Health services research

## Abstract

Public health research is complex, involves various disciplines, epistemological perspectives and methods, and is rarely conducted in a controlled setting. Often, the added value of a research project lies in its inter- or trans-disciplinary interaction, reflecting the complexity of the research questions at hand. This creates specific challenges when writing and reviewing public health research grant applications. Therefore, the German Research Foundation (DFG), the largest independent research funding organization in Germany, organized a round table to discuss the process of writing, reviewing and funding public health research. The aim was to analyse the challenges of writing, reviewing and granting scientific public health projects and to improve the situation by offering guidance to applicants, reviewers and funding organizations. The DFG round table discussion brought together national and international public health researchers and representatives of funding organizations. Based on their presentations and discussions, a core group of the participants (the authors) wrote a first draft on the challenges of writing and reviewing public health research proposals and on possible solutions. Comments were discussed in the group of authors until consensus was reached. Public health research demands an epistemological openness and the integration of a broad range of specific skills and expertise. Applicants need to explicitly refer to theories as well as to methodological and ethical standards and elaborate on why certain combinations of theories and methods are required. Simultaneously, they must acknowledge and meet the practical and ethical challenges of conducting research in complex real life settings. Reviewers need to make the rationale for their judgments transparent, refer to the corresponding standards and be explicit about any limitations in their expertise towards the review boards. Grant review boards, funding organizations and research ethics committees need to be aware of the specific conditions of public health research, provide adequate guidance to applicants and reviewers, and ensure that processes and the expertise involved adequately reflect the topic under review.

## Background

When writing and reviewing research proposals in the area of health and the life sciences reviewers usually expect a clear-cut research question and/or hypothesis, a sound, straightforward, and well-proven methodology, well-defined outcome parameter(s) and a well-controlled setting which eliminates potentially interfering factors. Ethical review boards often expect informed consent or measures for continuous monitoring of each individual study participant. Furthermore, the proposal should be based on one single established theory or model. Public health topics, instead, usually feature less controllable settings. They cover diverse populations, demand multifaceted observational approaches or interventions, and often involve different epistemological perspectives. For example, obesity, arguably one of today’s most important public health topics, is related to societal factors such as cultural norms, the natural and the built environment (transport facilities, walkability), the setting (school, workplace, unemployment), the availability of and advertising for foods, as well as individual physical (calorie-intake, exercise), psychological (stress, coping behaviour) and social factors (income, educational level, family, friends), and genetic predispositions [1]. Cluster-randomized trials or pragmatic trials on public health interventions for obesity might include unique features that complicate the application of standard guidelines for ethics review [2, 3]. Depending on one’s epistemological viewpoint, obesity will be framed as an epidemic, a social construction, a symptom for a dysfunctional society, individual misbehaviour, a genetic condition, or all of these.

According to the definition of WHO, public health *“…refers to all organized measures (whether public or private) to prevent disease, promote health, and prolong life among the population as a whole. Its activities aim to provide conditions in which people can be healthy and focus on entire populations, not on individual patients or diseases. Thus, public health is concerned with the total system and not only the eradication of a particular disease*” [4]. For the purpose of this article we understand public health in the same broad way, i.e. referring to organized health-related measures that focus on populations, not on individual patients. Health systems research, health services research, health technology assessment, and similar research streams are thus explicitly included, as well as medical sociology or anthropology and applications of similar disciplines to health research. In contrast, clinical research, focusing on individual patients, is not considered here.

In relation to the challenge of obesity, there has been a large array of projects comprising narrowly defined interventions, patient groups and outcomes, which were conducted in a highly controlled setting and used only one theory and one method. Dieting for weight loss in well-defined groups is one example. While some of these projects delivered valuable insights, others generated rather inconsequential results [5]. If, however, the reach and effectiveness of an intervention depend largely on its implementation in a specific context, public health research needs to take these factors and the complex interactions between them into account in order to be meaningful. It often needs to integrate different methods, and may for example combine (quantitative) epidemiological data with data from (qualitative) narrative interviews. It embraces multiple disciplines as distinct as medicine, economics or cultural anthropology, uses inter- and trans-professional approaches and is nurtured by multiple epistemological and theoretical streams: a social constructivist perspective may thus be triangulated with critical rationalism [6, 7]. Dealing with this kind of multidimensionality is a constant challenge for researchers/applicants, referees and review boards assessing the quality, relevance and ethics of public health research.

### Public health research at the German research foundation (DFG)

This challenge has also been felt by applicants, referees, and review boards of the DFG, the largest independent research funding organization in Germany. Over the past decades, few interdisciplinary public health research proposals have been received and funded. While the acceptance rates for proposals in any health field do not differ widely at the DFG (currently about 35%), the average funding awarded to projects in public health is considerably smaller as compared to funding of biomedical projects. Furthermore, most of the projects funded within the subject area “Public Health, Health Services Research, Social Medicine” were concerned with technical (as opposed to social) topics and focused on the investigation of mono-causal, linear relationships. Interdisciplinary proposals, projects using qualitative or mixed methods, or more applied interventional public health research were almost absent, although there has been a slight increase in the last years [8].

### Aims and approach

In January 2014, the DFG and members of the scientific public health community serving at various boards of the DFG initiated a round table discussion on challenges and possibilities in applying for and reviewing public health research proposals. As it was assumed that this problem was neither specific to the DFG nor for the situation in Germany, researchers and representatives of other funding organizations from Germany, the United Kingdom, Switzerland, and the Netherlands were also invited. The participants were decided upon by a steering committee consisting of the members of the DFG review boards (who are elected by their research communities) and the DFG head office. Further suggestions for other participants were taken up in due course. All 35 participants had much experience in writing as well as reviewing the quality and ethics of public health proposals, and care was taken to have a broad spectrum of methodological and topical expertise represented. Additionally, participants with experience in the rating, prioritization and decision making of grant proposals were invited. The aim was to analyse the specific difficulties and to develop guidance to applicants, referees, review board members and funding organizations. This guidance should complement, not replace generic requirements of the DFG or other funding organizations. Ultimately, the aim is to facilitate and fund the most promising and innovative proposals both from a scientific and societal point of view. The scope of our guidance does not include the complementary and broad spectrum of ethical and regulatory issues because of recent guidance issued and ongoing initiatives for harmonized guidance [2, 3, 9, 10].

At the 2-day meeting, experts from the various countries presented on the respective structures and processes related to reviewing and funding public health research, and on its current situation and methodological challenges [11]. A core group of participants (the authors of the present paper) were commissioned to summarize results into suggestions for writing and reviewing public health research proposals, with relevance for Germany and beyond. The draft underwent several rounds of revisions by the authors until consensus was reached.

The article addresses two levels: more generally, issues related to complexity, interdisciplinary research, ethical implications and different epistemological perspectives are considered. Quantitative methods/epidemiology, qualitative methods and mixed methods are explicitly discussed, referring to relevant literature or instruments for more detailed, specific guidance.

## Review

### Challenges in writing and reviewing the relevance and quality of public health research

Public health research differs from laboratory, clinical and psychological research, which often investigates narrowly-defined hypotheses under standardized and controlled experimental settings. Applying the criteria of the latter to public health research may lead to problems in the process of reviewing proposals that attempt to build bridges between disciplines by combining different research paradigms.

#### Complex interventions, real-life settings and controllability of studies

The United Kingdom Medical Research Council (UK-MRC) defined several dimensions of complex interventions. These dimensions include “*the number of interacting components* […]*, the number and difficulty of behaviours required by those delivering or receiving the intervention* […]*, the number and variability of outcomes* […]*, the degree of flexibility or tailoring of the intervention permitted*” [12]. It is suggested that complexity can be induced by (1) the intervention itself (composed by several qualitatively different and interacting components), (2) the context in which the intervention will be implemented and (3) in the interaction between intervention and context; and (4) that complexity may also result from the fact that the intervention – not well defined at the beginning – will initiate a learning system which creates a suitable structure for an effective change [12].

Faced with complex settings or interventions there are two types of error when designing a research project: it can oversimplify the situation or it can become too complex. Many applicants and reviewing bodies tend to prefer studies in well-controlled settings in which the efficacy of an intervention or its individual components can be tested with high internal validity. While studies can deliver valuable insights, they sometimes simplify a situation at the expense of generalizability. Therefore, public health projects often require navigating a middle way between internal and external validity when seeking relevance. Dealing with obesity is one example. Another example are the Sure Start Local Programmes (SSLPs) in England, which aim at improving the health and well-being of 3-year-old children. In the paper evaluating the intervention, the SSLPs are described as not having “*…a prescribed set of services, especially not those delineated in a manualized form to promote fidelity of treatment to a prescribed model. Instead, each local programme was responsible for working with the community to improve existing services according to local needs while covering core services…*” [13]. Altogether 14 outcomes were studied, among them the status of immunizations in children or child accidents, but also (from the child’s health perspective) intermediary outcome parameters such as the father’s interaction with the child and the mother’s rating of the local area [13]. In short, we are faced with loosely-defined, flexible interventions, highly heterogeneous modes of implementation and context, and multiple intermediary and final outcomes. With respect to these issues, the UK-MRC guidance on developing and evaluating complex interventions as well as a more recent MRC-guidance on process evaluation emphasize the importance of a good theoretical understanding and of distinguishing between implementation failure and genuine ineffectiveness [12, 14]. These guidelines highlight the need to assess a range of measures rather than a single outcome parameter, and to use an adaptive rather than a rigid protocol. Obviously, such an approach leads to multiple methodological challenges: How can we define interventions, modes of implementation, and context in a way that reflects reality (or better, realities) and is operational and sufficiently rigorous at the same time? How can multiple outcomes be analyzed and interpreted in a systematic and transparent way? What is the relationship between intermediary and ultimate outcomes? Researchers and reviewers alike need to be aware of, accept and assess the trade-off between generating results that are relevant and rigorous research methodology. While interventional clinical researchers are mostly used to highly standardized and controlled settings and interventions, interventional public health research may require adaptation throughout the project. This is not necessarily a methodological or ethical flaw; adaptive planning may rather become a quality criterion if justified appropriately.

#### Interdisciplinarity, expressed through different epistemological approaches

There is no unifying theory of public health. The combination of different disciplines and methods from distinct fields of science entails a substantial theoretical and epistemological heterogeneity. In the daily practice of public health this issue is often hidden or overlooked. To adequately address this challenge, public health research often has to adopt an inter- or transdisciplinary approach. Scientists from a multitude of disciplines may be part of one research team, for example, economists, geographers, psychologists, physicians, health services researchers, political scientists, or those of normative/axiological sciences. The art of joining these disciplines through the lens of a well-defined research question without becoming shallow is the foundation of the originality, innovation and added value of interdisciplinary and transdisciplinary research. Transdisciplinarity requires that researchers from different disciplines explore research questions “*…at the intersection of their respective fields, conducting joint research projects and …developing methodologies that can be used to re-integrate knowledge,* […] *promoting theoretical, conceptual, and methodological reorientation with respect to core concepts of the participating disciplines*” [15]. Reviewers evaluating public health proposals may tend to focus mainly on their own respective field and thus selectively notice deficiencies of interdisciplinary proposals rather than their strengths. Thus, mixed panels of reviewers with different backgrounds face the challenge of not coming up with a list of flaws according to each discipline, but rather judging projects in an integrative way.

Even the various disciplines involved in public health research need to deal with heterogeneity of their respective fields. For example, epidemiologists or analytically oriented sociologists would primarily explain the occurrence of outcomes by assessing how they are associated with exposures of interest, thus thinking in directional, one-dimensional pathways or mechanisms rather than complex, multi-dimensional or even circular models; they would apply relatively strict – albeit still subjective – guidelines for inferring causality, with the aim of explaining observations (D. Hume: “explain”). Anthropologists, on the other hand, might aim to grasp and understand human behaviour underlying a particular phenomenon by applying more abstract theories (W. Dilthey: “understand”). Thus, anthropologists might end up criticizing public health research proposals with a strong epidemiological component as being theoretically underdeveloped, whereas epidemiologists may perceive anthropological research as tending to over-interpret empirical data. If even more scientific disciplines are involved, evaluation requires expertise in multiple fields, plus the expertise and openness to adequately evaluate the combination of these. Reviewers who are experts for some aspects, and less so for public health in general, may have difficulties in appropriately judging the entire project. If this problem is to be resolved by involving more reviewers, this might lead to an even larger number of critical remarks. This can easily become prohibitive at the level of review boards – if many proposals from very heterogeneous disciplines (including experimental biomedical research) must be judged at a time and with restricted financial resources at hand, simple heuristics based on number and strength of critical remarks may prevail. Reviewing and deciding on complex public health projects thus requires considerable latitude and the ability to integrate across several thematic fields and methods, and the capacity to apply rigorous quality criteria in an integrative rather than aggregative way.

#### Interdisciplinarity, expressed through different methods

Using different methods is characteristic of public health research. If justified and employed appropriately, combinations of research methods stemming from quantitative and qualitative approaches should be considered a particular strength. Mixing of methods may be used towards four primary purposes: complementarity (methods used to address different aspects of the same question), expansion (methods used to address different questions), development (one method used to inform the development of the other), or confirmation (where the results of two methods converge), with the first three more widely used for pragmatic reasons [16]. Research methods stemming from qualitative and quantitative approaches may be applied side-by-side in distinct parts of a given study, or they may be employed in a truly integrated manner from study design through to data collection, analysis and interpretation of findings (see section on Mixed methods research). No single reviewer will be familiar with all suitable methods stemming from different quantitative or qualitative methodologies, not to mention all combinations of them. However, in order to make integrated judgments of interdisciplinary proposals, reviewers need to have at least a basic understanding of the requirements related to different methods. Applicants should adhere to certain standards of describing their methods, and, very importantly, they should justify clearly why they chose specific (combinations of) methods and why their choice constitutes the most appropriate approach given the question at hand. In the following, we provide an orientation for describing and reviewing proposals employing epidemiology (as the core methodology for quantitative methods in public health), qualitative methods and mixed methods approaches. While this distinction provides some orientation, it should be kept in mind that quantitative and qualitative research methods do not simply represent two different research paradigms as there can be a substantial overlap between methods used [17] and that different methodologies or even epistemologies may exist within the qualitative field [18].

#### Quantitative methods/epidemiological designs

Epidemiology continues to be a core discipline of public health, providing quantitative data on the population distribution of health, disease and their determinants, and on the associations between specific health outcomes and risk or protective factors as well as preventative or curative interventions. Depending on the field in which epidemiological methods are applied, such factors can be found at the individual or regional/societal levels. Observational studies are a core approach used in epidemiological scientific enquiry, as the population exposures and risk factors of interest generally cannot be allocated randomly to groups, but rather are assessed in relation to concomitant changes in health endpoints. They continue to provide major contributions to knowledge on risk and protective factors for a broad range of health conditions worldwide. The value of modern observational studies lies in this opportunity to link more and more detailed individual and contextual risk factor information with well-defined health outcomes. At the same time, a rich theoretical development took place to understand confounding and bias in observational research and to provide strategies for dealing with these. As an example, the largest-ever prospective observational study in Germany, the National Cohort Study aiming to involve about 200,000 participants, was launched in 2014 to strengthen the understanding of causes of common disorders such as cancer and cardiovascular diseases [19]. Together with planning and implementing the National Cohort Study, tailored policies for informed consent and data protection were developed to address concerns of research ethics committees. Interventional studies in public health research comprise randomised and non-randomised approaches. Examples for randomised controlled trials in population-based intervention studies are the U.S. Physicians’ Health Study [20] or a study on specific health effects of improved stoves in developing countries [21]. Cluster randomised controlled trials or community interventions are more frequently used in public health research as randomisation (and thus informed consent) at individual level is often not feasible [2, 22]. However, also for ethical reasons, not all public health measures can be evaluated prospectively and administered only to a limited group, while maintaining a control group without the intervention. In these cases, further designs, including quasi-experimental studies (where allocation to interventions is not per random assignment), controlled before–after studies (where observations are made before and after an intervention is implemented, with the use of a control group), as well as time series designs (where observations over multiple time points are made) and evaluations of natural experiments (using, for example, policy changes as interventions and applying the methods above and/or a range of statistical approaches) are accepted research tools, as for example suggested by the UK-MRC Guidance [23] and the Cochrane Collaboration’s EPOC group [24].

#### Qualitative methods

Qualitative methods have been considered essential to public health research for quite some time [16]. They play a critical role in furthering and deepening our understanding of the social and broader causes of a problem and in designing public health interventions and implementation mechanisms that are appropriate and acceptable to the target population and therefore likely to be effective. Qualitative methods offer particular strengths in the analysis of lay persons’ or professionals’ perceptions on health-related issues, in understanding health-related issues within a biographical perspective, in the handling of complexity of variables and their interactions, as well as in the integration of contextual conditions [25]. These reasons also comprise analysis of interactions (e.g. patient-professional, team), organizational issues, power relationships and their expressions in the design, conduct and implementation of a public health intervention. The strength of qualitative analysis is to move beyond the apparent or manifest by elaborating latent characteristics or explications through the researchers’ interpretation. However, ‘qualitative’ does not resort to a set of fixed methods or designs. It is an umbrella term for a variety of social research approaches, which are founded – more or less – on the ideas of openness, subjectivity, interpretation of meaning and process orientation [26]. The idea of openness relates to various fundamental aspects of research, including the research process and methodological decisions, or the researcher’s stance towards the research subjects and the types of questions asked. For example, in a study of the practice of informed consent, researchers had to be sensitive to the idea of different conditions, in which informed consent prior to surgery might even harm the experience of autonomy in patients [27].

Subjectivity relates to the idea of the researcher always being part of the research process and therefore the need of a continuous reflection of how one’s own assumptions, feelings, conceptions, etc. shape research decisions and study results. This is of special importance to qualitative work, since the core of qualitative work is interpretation of meaning, or reconstructing the meanings persons develop to understand the world they live in. In contrast to quantitative research, where the process is mostly linear and deductive, qualitative research designs and processes are rather circular or iterative and often inductive. For example, theoretical sampling following a grounded theory or comparative case study approach uses results from the analysis of the first case(s) to reflect on criteria to sample the next cases to be analysed.

Main modes of inquiry in qualitative public health research are individual interviews, group discussions (often called focus groups), observations on individuals, groups, organizations or geographical units, either by participating or shadowing or non-participatory, with ethical challenges regarding informed consent standards but with the potential to analyse what is often not recognized, deemed self-evident or socially unacceptable, and document analysis, working with data that has not been constructed as part of a research project but of natural conduct, e.g. reports on government programmes, minutes of meetings, medical records, or homepages. Guidance for ethics review of qualitative research is less harmonized than for quantitative studies, but helpful information can be found, for example, from the United Kingdom Economic and Social Research Council [28].

#### Mixed methods

Mixed methods research has been employed in public health and health services research for several decades. Rooted in a pragmatic approach, where a paradigm is defined as a set of shared beliefs in a given research field that determines which questions are most meaningful and which procedures are most appropriate for answering those questions, combining quantitative and qualitative methods in a fruitful way is nowadays considered possible [29]. Mixed methods research has been defined as research that bridges and integrates (1) qualitative and quantitative research questions, (2) qualitative and quantitative research designs, (3) qualitative and quantitative techniques for collecting and analysing data, and (4) qualitative and quantitative findings [30]. In doing so, it allows flexible combinations of one or several qualitative and research components. Through integration, the resulting insights foster a more holistic and in-depth understanding and go beyond a simple additive combination of results obtained through quantitative and qualitative components.

The application of mixed methods research is particularly appropriate where complex public health phenomena are concerned, whether in relation to assessing and understanding the causes of a problem, conducting formative research to develop and test interventions, or evaluating the impact of a large-scale implementation of technical, programmatic or policy interventions. Using questions related to the effectiveness of public health interventions as an example, mixed methods research can be applied to:

- Add explanation to insights gained from quantitative evaluations, for example, by furthering the understanding of how and why an (effective) intervention operates
- Provide pointers to re-designing or adapting interventions, for example, where a quantitative evaluation has shown that an intervention is underperforming or ineffective
- Increase the methodological returns from cost- and labour-intensive evaluations in terms of knowledge related to instrument and measure validation and core constructs
- Generate knowledge on how to bridge the research–policy/practice divide, for example, by providing evidence on how to best implement an intervention

Importantly, mixed methods research can be conducted at the primary research level as well as at the level of systematic reviews [31, 32]. There are some common pitfalls, namely issues of poor reporting, inadequate justification for use, and poor integration of methods. Practical challenges include the repeated underfunding of qualitative components [33].

### Implications for applicants, reviewers and review boards of public health research

Applicants need to keep in mind that not all reviewers will be familiar with all theories and methods employed in the project. Therefore, they should sketch the full complexity of the topic they aim to investigate, explain the theories, models and assumptions their project is based on, be explicit about why certain methods are chosen, and be transparent regarding their potential and limitations, as well as contingencies. Box 1 summarizes aspects to be considered when writing public health proposals.

**Box 1:** Summary points for those writing public health research proposals

• The theory/theories, the model(s), and the assumptions need to be made explicit, as well as the rationale of focusing on these (and not on others) • The research question needs to be developed and described against this background • The potential implications for policy and practice need to be explained in a plausible way • With reference to theories/models/assumptions, the complexity of the topic needs to be outlined in enough detail so that the choices of the research question, the population, the intervention (if any) and the outcomes become comprehensible • The choice of methods (or of the combination of methods) needs to be described in the light of the underlying theories/models/assumptions and the research question; this includes assessment/justification of quantitative data sets to be generated or used, or methods of sampling, inquiry and analysis for qualitative data • Explicit reference to existing guidelines for conducting high-quality research should be made. For epidemiological research, these include, for example, guidelines on good epidemiologic practice by the International Epidemiological Association [34] or the German Society for Epidemiology [35]. For qualitative research, relevant tools include guidance on managing the quality of qualitative research as described by the United Kingdom Government Chief Social Researcher’s Office and in a well-known textbook by Flick [36, 37]. For mixed methods research, the Mixed Method Appraisal Tool [38] and the guidance for writing and reviewing proposals using mixed methods, commissioned by the Office of Behavioral and Social Sciences Research of the United States National Institutes of Health [39] provide helpful information • It should be noted that the challenges of inter- and transdisciplinary research are often not sufficiently addressed by these tools and guidelines • In terms of reporting, research projects should be expected to consider the respective reporting guidelines where these apply (for details see [40]). For qualitative research, the Consolidated criteria for REporting Qualitative research) [41] has been proposed; for mixed-methods research, Good Reporting of A Mixed Methods Study [42] represents an attempt to ensure good reporting	

Reviewers need to be open to theories and methods they are not familiar with. They should check if the choice of theories, the research question and the methodology is well justified (i.e. based on the references and the reasoning of the applicants) and in accordance with the respective ethical guidelines [2, 28]. They should be reflective regarding the trade-off between methodological purity and relevance. They should also be aware of the additive and exponential effects of critical remarks in interdisciplinary projects and clearly state if their remarks are suggestions for improvements or represent serious concerns. More details are presented in Box 2.

**Box 2:** Summary points for reviewers of public health research

• Have the theoretical background/the underlying model and assumptions been made explicit? Are they adequate for the topic? Please keep in mind that there is always more than one theory/model that will be adequate. If your preference deviates from the applicants’ preference: is this a major flaw of the project or within an acceptable range? To what extent is the choice of the theory/model likely to lead to flaws in the project? • Has the complexity of the topic been represented adequately? Does the (necessary) choice of the aspects the project will cover lead to an undue oversimplification? On the other hand, do you think that the project should be more focused, using less resources? If you think so, please explain why this would not jeopardize the aim of the project • Is the choice of methods well substantiated? If you think that more/less/other methods would be more appropriate please provide reasons. If you do not fully agree with the choice of methodology: does the choice of methodology make the project futile? Would another methodology have the potential to generate better results with a similar requirement of resources? If more resources are needed for the alternative methodology: would this still be efficient? If you suggest omitting a method: what consequences does this have for the main research question? If you are not familiar with certain methods, please state so. If you feel that your review would benefit from the exchange with other reviewers who are familiar with those parts of the methodology, please state so • Public health projects that reflect real life are usually full of contingencies. Have these contingencies been made explicit? Have the applicants explained how they will curtail risks and remedy potential deficiencies? Bear in mind that public health projects which reflect real life can often not be reduced to single hypotheses and require preparedness for dealing with new situations during the course of the project; these should not be reasons to reject or downgrade a proposal	

Review boards and funding organizations need to be aware of the particular needs and challenges of public health research and have these reflected in their guidance to applicants and reviewers. When monodisciplinary health research projects and public health research projects are directly or indirectly competing for the same funds, review board members should be especially aware of the likely accumulation of critical remarks in interdisciplinary projects. Further suggestions are summarized in Box 3.

**Box 3:** Summary points for review boards and funding organizations in public health research

• Depending on the composition of the review board, initiate discussions and reflections on public health research, its similarities and differences with clinical and laboratory research, and the resulting implications for the reviewing and decision process • Reviews on the often inter-/transdisciplinary proposals will usually be done by specialists in one discipline/method; if several disciplines and methods have been covered by different reviewers, encourage and moderate exchange among them • Be aware of the cumulative effect of critical remarks in reviews; the number of critical remarks is likely to increase with the number of theories and methods employed and the number of reviewers involved even if the quality of the project remains the same • There is a potential trade-off between internal validity and relevance; if a public health research question is stripped of its context the internal validity may be high but the relevance low. On the other hand, a low internal validity cannot lead to relevant results • Strengthening the ‘relevance-under-real-life’ perspective can also be achieved by including potential users of research-results in the review board • Projects comprising a combination of disciplines and methods can be expected to result in enhanced insights; however, they may require more resources than projects that are confined to one discipline and employ only one method	

## Conclusions

Public health research involves various disciplines, epistemological perspectives and methods, and is rarely conducted in a controlled setting. This has been identified as a challenge for applicants, reviewers and review boards alike. The aim of this article is to develop guidance to applicants, reviewers and funding organizations of public health research. Applicants should not overestimate the pre-existing knowledge of reviewers and review boards with respect to the theoretical background and the rationale behind formulating research questions and choosing certain methods in their specific field of enquiry. They should be prepared to describe in detail why they apply certain theories, why the research question is neither too narrow nor too broad, and why their specific method or combination of methods is the most appropriate in order to obtain valid and meaningful results.

Reviewers must be open to this multifaceted field of research and its specific challenges. They need to make the rationale of their judgments transparent while referring explicitly to the corresponding standards. They should accept and state their own limitations in assessing a proposal or certain parts of it and be prepared to suggest an exchange with other experts on proposals that are at the interface of different epistemologies and methods.

Review boards and funding organizations need to sensitize their reviewers to different research cultures, demand a corresponding openness and actively encourage their reviewers to explicitly verbalize the doubts or limitations they might have. Review boards should constantly reflect on the cumulative effect of critical remarks and possible tendencies of rating straightforward mono-disciplinary proposals above more complex, multifaceted ones. This is particularly important if, instead of having separate funding streams and review boards for individual research fields (for example, public health research), they are obliged to competitively evaluate and decide on proposals with very heterogeneous topics, methods and backgrounds.

While all these aspects are of high relevance for better writing and reviewing public health research, they should always be seen as part of – and not replacing – general scientific standards.
